# Heat stress modulates polymorphonuclear cell response in early pregnancy cows: I. interferon pathway and oxidative stress

**DOI:** 10.1371/journal.pone.0257418

**Published:** 2021-09-20

**Authors:** Carolina dos Santos Amaral, Gabrielle Rebeca Everling Correa, Lady Katerine Serrano Mujica, Mariani Farias Fiorenza, Suzan Gonçalves Rosa, Cristina Wayne Nogueira, Valério Marques Portela, Fábio Vasconcellos Comim, William Schoenau, Natalia Pavlovna Smirnova, Alfredo Quites Antoniazzi

**Affiliations:** 1 Biotechnology and Animal Reproduction Laboratory, Federal University of Santa Maria, Santa Maria, RS, Brazil; 2 Synthesis, Reactivity and Organocalcogens Pharmacological and Toxicological Assessment Laboratory, Federal University of Santa Maria, Santa Maria, RS, Brazil; 3 Independent Researcher, United States of America; University of Illinois, UNITED STATES

## Abstract

One of the major causes of early pregnancy loss is heat stress. In ruminants, interferon tau (IFNT) is the embryo signal to the mother. Once the interferon signaling pathway is activated, it drives gene expression for interferon-stimulated genes (ISGs) and alters neutrophils responses. The aim of the present study was to evaluate interferon (IFN) pathway, ISGs and gene expression in polymorphonuclear leukocytes (PMN) and oxidative stress in dairy cows under heat stress. Pregnant cows had their estrous cycle synchronized and randomly assigned to a comfort or heat stress group. Blood samples were collected at artificial insemination (AI) and on Days 10, 14 and 18 following AI. Pregnant cows were pregnancy checked by ultrasound on Day 30 and confirmed on Day 60 post-AI. Results are presented as mean ± SEM. The corpus luteum (CL) diameter was not different between groups of pregnant cows; concentration of progesterone of pregnant cows on Day 18 following AI was greater in comfort group compared to heat stressed group. Comfort pregnant cows had higher expression of all analyzed genes from interferon pathway, except for *IFNAR1*, on both Days 14 and 18. Conversely, heat stressed cows did not show altered expression of IFNT pathway genes and ISGs between Days 10, 14, and 18 after AI. The oxidative stress, determined as malondialdehyde (MDA) levels, was greater in heat stress group on Days 10, 14 and 18, independent of pregnancy status. Heat stress negatively influences expression of ISGs, IFN pathway gene expression in neutrophils, and oxidative stress. Our data suggest that lower conception rates in cows under heat stress are multifactorial, with the association of interferon pathway activation and the unbalanced oxidative stress being main contributing factors.

## Introduction

Pregnancy loss is an important factor that reduces reproductive performance in dairy herds, and it occurs more frequently in the first 30 days of pregnancy [1, 2]. One of the major causes of pregnancy loss and implantation failure during early pregnancy is heat stress (HS) caused by the increase of temperatures during summer (hot seasons) [3, 4]. Effects of HS on follicular development, steroidogenesis, quality of gametes and embryos are well studied [5–9]. HS may also be an end result of an imbalance between reactive species production and antioxidant capacity, which leads to oxidative stress [10–12].

Maternal recognition of pregnancy in ruminants occurs between Days 10–20 of pregnancy, when the conceptus signals its presence in uterus [13]. Interferon tau (IFNT) is the major cytokine responsible for the embryo-maternal interaction to avoid luteolysis [14, 15] and to establish and maintain the pregnancy [16]. It is produced by embryonic trophoblast cells at pre-implantation period and acts in the endometrial luminal epithelium in a paracrine manner. IFNT inhibits endometrial estrogen and oxytocin receptors expression and prevents prostaglandin F2 alpha (PGF) luteolytic pulses [17]. IFNT also acts in extrauterine tissues (e.g. leukocytes and corpus luteum) [18, 19], protecting the corpus luteum (CL) [20, 21] and signaling the early pregnancy to peripheral blood cells via induction of interferon stimulated genes (ISGs) [22, 23].

In general, it is believed that concentrations of IFNT circulating in the bloodstream are extremely low and thus difficult to detect [19, 21]; however, there are few indirect approaches to detect IFNT action in the bloodstream. One of them is to measure ISGs expression in leukocytes. There are several studies that correlate ISGs expression in peripheral blood leukocytes (PBL) with early pregnancy [22–25]. These studies have analyzed gene expression in separate PBL fractions, especially the polymorphonuclear leukocytes (PMN), which are most sensitive to IFNT stimulation, when compared to mononuclear cells fraction (PBMC). Additionally, there is a positive correlation between ISGs expression in PMN and early pregnancy in cattle [26, 27]. Although IFNT is not virus inducible [28], it belongs to type I IFNs and uses the same mechanism of action of antiviral response genes. IFNT binds to type I IFN receptors (IFNAR) 1 and 2 [29, 30] and activates the Janus kinase-signal transducer and activator of transcription (JAK/STAT) intracellular pathway [31]. Subsequently, phosphorylated STATs dimerize and recruit IFN-regulatory factor 9 (IRF9) to form STAT1-STAT2-IRF9 tri-complex (interferon-stimulated gene factor 3, ISGF3). This complex translocates into the nucleus to initiate transcription of ISGs [32].

Pregnancy causes an immunological challenge because a semi allogenic fetus must be supported within the pregnant female for the required gestational period. The decidua and placenta of human and mouse form key immunological barriers that sustain maternal tolerance, yet generate innate immune responses that prevent microbial infections [33]. The biology underlying the systemic crosstalk of early embryo signaling and immune system is not completely understood. Therefore, IFNT endocrine action may alter immune cells response during early pregnancy.

Considering the low pregnancy rates during warm season and the endocrine signaling of IFNT characterized by ISGs expression in extrauterine tissues, we hypothesized that oxidative stress caused by heat stress negatively impacts progesterone production and innate immune response during early pregnancy in dairy cows. The objective of our study was to evaluate relations between concentration of progesterone, oxidative stress blood markers, expression of ISGs and genes of IFN signaling pathway in neutrophils of dairy cows under comfort or heat stress environment on embryo pre-implantation period. We tested whether high temperatures during summer affect the ability of the pregnant dairy cows to signal the embryo presence and modulate IFN pathway.

## Materials and methods

### Chemicals

Unless otherwise indicated, chemicals and reagents were purchased from Sigma Chemical Company (Sigma-Aldrich, St. Louis, MO, USA).

### Cattle and herd management

The study was approved by the Animal Care Use and Committee (CEUA-UFSM # 5728120217) of Federal University of Santa Maria and conducted on a commercial dairy farm in Southern Brazil. Thirty-two multiparous Holstein dairy cows in lactation from the same herd were included in this study. The cows were 3 to 6 years old, body condition score greater than 2.5 (1 = thin and 5 = obese in a scale 1 to 5), absent of any detectable reproductive and clinical disorders during the study period. Cows were milked twice a day and fed complete ration and corn silage, with *ad libitum* access to water. All sampling and data collection for this study were obtained with no additional distress.

### Experimental design, synchronization protocol and Artificial Insemination (AI)

The experiment was conducted during two distinct seasons. The samples from comfort cows group (n = 15) were collected in September (Late Winter/Early Spring), when the temperature-humidity index (THI) is approximately 65–70 in Southern Brazil. The samples from the heat stressed cows group (n = 17) were collected in January (Summer), characterized by high temperatures associated with high humidity, when THI is approximately 80–85. Both groups had their estrus synchronized with the same protocol [34]. The estrous cycle synchronization protocol was initiated by the insertion of an intravaginal device (IVD) containing 1.9g of progesterone (CIDR, Zoetis, São Paulo, Brazil), administration of 2mg (i.m.) of estradiol benzoate (Sincrodiol, Ourofino, Minas Gerais, Brazil) and 2mL (i.m.) of gonadorelin, an analogue of GnRH (Cystorelin, Boehringer Ingelheim, São Paulo, Brazil) 11 days prior to AI (Day -11). Four days before AI (Day -4) the first injection of 0.5mg (i.m.) of sodium cloprostenol, a synthetic prostaglandin F2 alpha analogue, was administered (PGF; Sincrocio, Ourofino). Two days before AI (Day -2), IVD was withdrawn and the animals received the second injection of 0.5 mg (i.m.) of PGF and 1mg (i.m.) of estradiol cypionate (ECP; E.C.P. Zoetis). Only animals that exhibited standing estrus by 48 hours after IVD withdrawal were included in the experiment (Comfort cows group n = 12; Heat Stressed cows group n = 13). AI was performed 48 hours (Day 0) after IVD withdrawal, using conventional semen. The semen was obtained from ST genetics® commercial company, stored in liquid nitrogen, and thawed at 36°C for 30 seconds for subsequent AI.

### Physiological parameters and environmental data

Respiratory rate (RR), heart rate (HR), and rectal temperature (RT) were evaluated at 3 p.m. on Days 10, 14 and 18 following AI. RR was expressed in breaths per minute (bpm) and was obtained using a timer to count respiratory movements for 30 seconds, multiplied by 2 to obtain the number of breaths per minute. HR was expressed in beats per minute (bpm) and was obtained using a flexible stethoscope (Standard, Bic Med, São Paulo, Brazil) placed directly into the left thoracic region under one of the auscultation foci for 30 seconds, multiplied by 2 to obtain the number of heart beats per minute. RT was measured with a large animal clinical thermometer inserted at 3 cm depth into the rectum and held to maintain contact with the mucosa for one minute. Body condition score (BCS) was determined at the beginning of the experimental period (estrus synchronization) and weekly throughout the study. A scale of 1 (thin) to 5 (obese) in increments of 0.25 units was used, as described by Ferguson, Galligan [35]. A single observer evaluated the BCS throughout the study to minimize variations. Ambient temperature and relative humidity (RH) were recorded at 4 p.m. on Days 0, 10, 14 and 18. The THI was calculated using the mathematical equation [36]: THI = (0.8 × Dbt) + [(RH/100) × (Dbt – 14.4)] + 46.4; where Dbt = dry bulb temperature, and RH = relative humidity.

### Blood sample collection

Blood was collected from the coccygeal vein using a 21G needle coupled to a vacuum collection system (BD Vacutainer®) into 4 mL EDTA-containing tubes. The collections were performed at the time of AI (Day 0) and on Days 10, 14 and 18 following AI. Blood was obtained in two tubes of 4 mL containing EDTA for each experimental time point. The first 4 mL tube of blood was used for oxidative stress assays and the second tube for isolation of blood leukocytes and determination of blood concentration of progesterone.

### Isolation of polymorphonuclear (PMN) peripheral blood cells

Isolation of PMNs was performed as follows. Briefly, after blood collection, 2 mL of whole blood was diluted in equal volume of 0.9% NaCl, followed by addition of 3mL of Ficoll-Paque PREMIUM®. Centrifugation was performed at 400xg for 15 minutes at room temperature. After centrifugation, the following layers were obtained: PBMC, Ficoll-Paque, PMN, and erythrocytes. All the upper fractions were withdrawn to collect the PMN fraction. PMNs were collected from the lower red layer. PMN samples were stored in a cryotube at -80°C for subsequent total RNA extraction. After isolation of PMN fraction, a glass-slide fraction-film was prepared to determine the purity of each fraction. Slides were stained using a rapid stain (Diff-Quik Differential Stains Set; Fisher Scientific, Waltham, MA, USA) according to the manufacturer’s recommendations. The cell fraction purity was accessed based on cell morphology. PMNs are classified as neutrophils, eosinophils, and basophils. They have condensed, segmented nuclei and are identified by the staining characteristics of their secondary granules. An experienced clinical pathologist examined the slides. A differential cell count was done by identifying 100 consecutive leukocytes using a 100x objective. Samples above 95% of specific cell type (PMN) [26] were included in this study.

### RNA extraction, reverse transcription, and real-time PCR

Total RNA was extracted from the PMN cells using Tri Reagent (BD), according to the manufacturer’s recommendations. Quantification and estimation of RNA purity was performed using Nanodrop spectrophotometer (Thermo Scientific, Waltham, MA, USA; RNA concentration mean 658.17 ng/μl, SD 226.61, minimum 225.7 ng/μl and maximum 999.1 ng/μl; Absorbance 260/280 nm ratio mean 1.91, SD 0.053, minimum 1.8 and maximum 2.01). RNA was treated with DNAse Amplification Grade (Thermo Fisher, Waltham, MA, USA) for 15 minutes at 27°C to degrade any DNA molecules. DNAse was inactivated with 1 μl EDTA for 10 minutes at 65°C. Reverse transcription was performed using iScript cDNA synthesis Kit (BioRad, Hercules, CA, USA) for 5 minutes at 25°C followed by 30 minutes at 42°C and 5 minutes at 85°C. Quantitative polymerase chain reaction (qPCR) was conducted in a thermocycler (BioRad, Hercules, CA, USA) using cDNA, forward and reverse bovine specific primers and SYBR fluorophore GoTaq® Green Master Mix (Promega Corporation, Madison, USA). The final reaction volume is 10 μl: 2 μl of cDNA and 8 μl of MIX (5 μl of SYBR, 1 μl of primer forward, 1 μl of primer reverse and 1 μl of water). Amplification was performed with initial denaturation at 95°C for 5 minutes followed by 40 cycles of denaturation at 95°C for 15 seconds and annealing/extension at 60°C for 30 seconds. To optimize the RT-qPCR assays, serial dilutions of cDNA templates were used to generate a standard curve, and efficiency between 90 and 110% was considered. Samples were run in duplicate and the results of expression of all analyzed genes were expressed by ΔΔCq method, having *GAPDH* and *RPS18* as reference genes, as previously described [37]. The genes assessed in this study are presented in Table 1.

**Table 1:** Primers designed for quantitative real-time PCR analysis.

Target	Primer sequence	GenBank
*ISG15*	F: GGTATCCGAGCTGAAGCAGTT	NM_174366.1
	R: ACCTCCCTGCTGTCAAGGT	
*MX1*	F: GTACGAGCCGAGTTCTCCAA	NM_173940.2
	R: ATGTCCACAGCAGGCTCTTC	
*MX2*	F: CTTCAGAGACGCCTCAGTCG	NM_173941.2
	R: TGAAGCAGCCAGGAATAGT	
*OAS*	F: GTGGCCAAAGGTGGCTCCTA	NM_001040606.1
	R: TGTGCCCAGATTTTGCTGAGG	
*IFNAR1*	F: GAATCAGCTCTACCCGCTAAT	NM_174552.2
	R: GCTCTGGCTTTGACACAATAC	
*IFNAR2*	F: AGCCAGAATGTGTCAGCGAT	NM_174553.2
	R: AGAACAGGCGCAACATACGA	
*STAT1*	F: CAAAGGAAGCCCCAGAGCCTA	NM_001077900.1
	R: ACATGCCACTCTTCTGTGTTCA	
*STAT2*	F: CAGCCCGTTTCAGGATCAGC	NM_001205689.1
	R: CAGTGCAGCTTTCTGCCAGT	
*JAK1*	F: GGGGTTAGCCGCTTAGGGAG	XM_024989564.1
	R: CCATTCAGAGCTGAGCACTTCC	
*IRF9*	F: GGTTCCTGAGATCGGCCACA	NM_001024506.1
	R: CCTGATTGAGCGGGGACAGT	
*GAPDH*	F: GATTGTCAGCAATGCCTCCT	NM_001034034.2
	R: GGTCATAAGTCCCTCCACGA	
*RPS18*	F: CCTTCCGCGAGGATCCATTG	XM_024983403.1
	R: CGCTCCCAAGATCCAACTAC	

F: Forward; R: Reverse.

### CL diameter and progesterone analysis

Corpora lutea diameter (mm) was measured on Day 18 following AI through ovarian ultrasonography (Mindray DP10 with a 6.5 MHz linear transducer). The concentration of progesterone was determined in plasma by chemiluminescent assay kit (ADVIA Centaur, Siemens) also on Day 18 following AI. Samples were run in duplicate and were analyzed at the same plate. The intra-assay coefficient of variation was 2.0% for progesterone.

### Malondialdehyde (MDA) levels

The determination of MDA concentration was performed as previously described [38]. Briefly, NaOH 3M was added to each sample, followed by incubation at 60°C for 30 minutes. After that, 6% H_3_PO_4_ and 0.8% thiobarbituric acid (TBA) were added to the system and the mixture was heated at 90°C for 2 hours. Then 10% SDS and n-butanol were added to extract the TBA-malondialdehyde (MDA) product, which was analyzed on Shimadzu® HPLC equipment. The analytical column was a Phenomenex® ODS-2 C_18_reverse-phase (250mm × 4.6mm, 5μm; 100Å, Allcrom, BR) and the mobile phase was ultrapure water and methanol (50:50; v/v). The HPLC analysis was performed under isocratic conditions at a 0.6 mL/min flow rate and UV detector set at 532 nm with a 20 μl sample volume injection. The results were expressed as nmol MDA/mg protein.

### Catalase (CAT) activity

The CAT activity was spectrophotometrically assayed by monitoring the H_2_O_2_ consumption at 240 nm [39]. The enzymatic reaction was performed by adding the sample and the substrate (H_2_O_2_) at 0.3 mM in a medium containing 50 mM phosphate buffer, pH 7.0. The enzymatic activity was expressed in units (1U decomposes 1μmol of H_2_O_2_/min at pH 7.0 and at 25°C)/mg protein.

### Superoxide dismutase (SOD) activity

The SOD activity was measured spectrophotometrically [40]. This method is based on the capacity of SOD to inhibit autoxidation of epinephrine to epinechrome. In this assay, each sample was diluted 1:10 (v/v) in phosphate saline buffer (PBS) and added in a 50 mmol/L Na_2_CO_3_ buffer pH 10.3 and the enzymatic reaction was initiated by adding epinephrine. The colorimetric reaction was measured at 480 nm and the results were expressed in units (1U decomposes 1μmol of epinephrine/min at pH 7 and at 25°C)/mg protein.

### Protein quantification

The protein concentration for MDA levels and CAT activity was measured by the method described by Bradford (1976). Protein concentration was determined using bovine serum albumin (BSA; 1 mg/ml) as the standard [41].

### Pregnancy diagnosis by ultrasound scanning

In both groups, uterine conditions were ultrasonographically evaluated using a Mindray DP10 with a 6.5 MHz linear transducer to select only animals without any evident pathology. Pregnancy rate was determined by dividing the number of pregnant cows at the pregnancy diagnosis at 30- and 60-days following AI by the total number of cows artificially inseminated (P/AI).

### Statistical analysis

Continuous data were checked for normality using Shapiro-wilk test. CL diameter, concentration of progesterone, physiological parameters, and productive performance data were analyzed by ANOVA followed by Student’s *t* test. Gene expression and oxidative stress data were analyzed by repeat measures for within-group analysis and standard least squares for between-group (comfort vs. heat stress and pregnant vs. non-pregnant cows) analysis. The main effects of day, pregnancy status (PS), treatment group, day by group interaction (day*group) or day by pregnancy status interaction (day*PS) were indicated. Differences of estrus occurrence and pregnancy were evaluated through chi-squared test. All data analysis was performed using the JMP7 Software (SAS Institute Inc., Cary, NC, USA). Results are presented as mean ± standard error of the mean (SEM) and are considered different at P<0.05.

## Results

### Cows in comfort or under heat stress environment: Physiological and reproductive parameters

**Fig 1:**
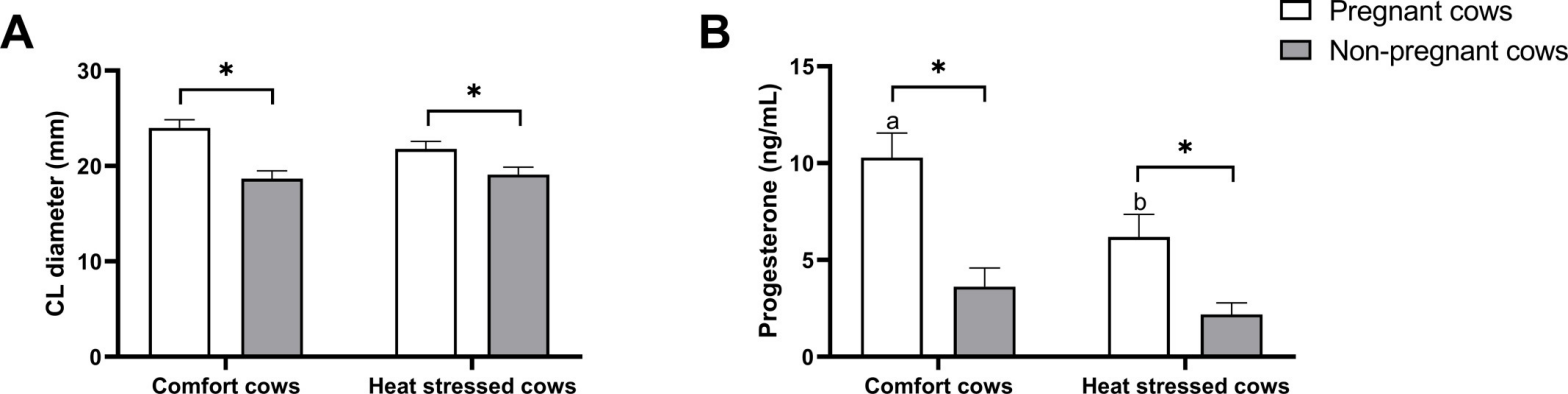
Ovarian corpus luteum diameter and concentration of progesterone in day 18 post-AI pregnant cows under comfort or heat stress conditions. A) Diameter (mm) of corpus luteum. B) Concentration (ng/mL) of circulating progesterone. Values are presented as mean ± S.E.M. Different letters represent significance at P<0.05 between comfort and heat stressed cows and asterisk represents significance at P<0.05 between pregnant and non-pregnant cows.

In order to determine the experimental model of heat stress, THI was calculated and the indices were different during summer and late winter/early spring in the experimental period (S1 Table). Thus, cows in the summer (higher THI) were considered under HS when compared to late winter/early spring (lower THI). HS affected RT, HR, and RR in dairy cows (P<0.05), which were evident at all timepoints (days along the season) (S1 Fig). Effect of season on estrous occurrence and pregnancy rate were not different between groups (P>0.05) and are presented in Table 2. Estrous occurrence rate was 80% (12 from 15 cows) in comfort group and 76.47% (13 from 17 cows) in heat stressed group. Pregnancy rate was 50% (6 from 12 cows) in comfort group and 38.46% (5 from 13 cows) in heat stressed group. CL diameter (Fig 1A) on Day 18 following AI was significantly different (P<0.05) in pregnant vs non-pregnant cows, when compared within-group, with larger diameter in pregnant cows independent of season. No differences in CL diameter in pregnant cows of the two groups were found (P>0.05). Concentration of progesterone followed the same pattern as CL diameter, however, it was lower in heat-stressed pregnant cows when compared to pregnant cows of the comfort group (P<0.05). In non-pregnant cows, the CL started to regress and, therefore, the CL diameter and concentration of progesterone did not differ between groups (P>0.05). In relation to milk production, cows were at similar days in lactation (S2A Fig), however, cows under heat stress had lower daily milk yield than the cows that were heat-not stressed (S2B Fig), confirming the experimental model.

**Table 2:** Effect of season on estrus onset and pregnancy rate.

Variable	N	(%)	P-value
**Estrus occurrence**			
Comfort cows	12/15	80.0^a^	p> 0.05
Heat stressed cows	13/17	76.5^a^	
**Pregnancy**			
Comfort cows	6/12	50.0^a^	p> 0.05
Heat stressed cows	5/13	38.5^a^	

Different letters represent significance at P<0.05 between comfort and heat stressed cows.

### Markers of oxidative stress in blood from cows in comfort or under heat stress environment

**Fig 2:**
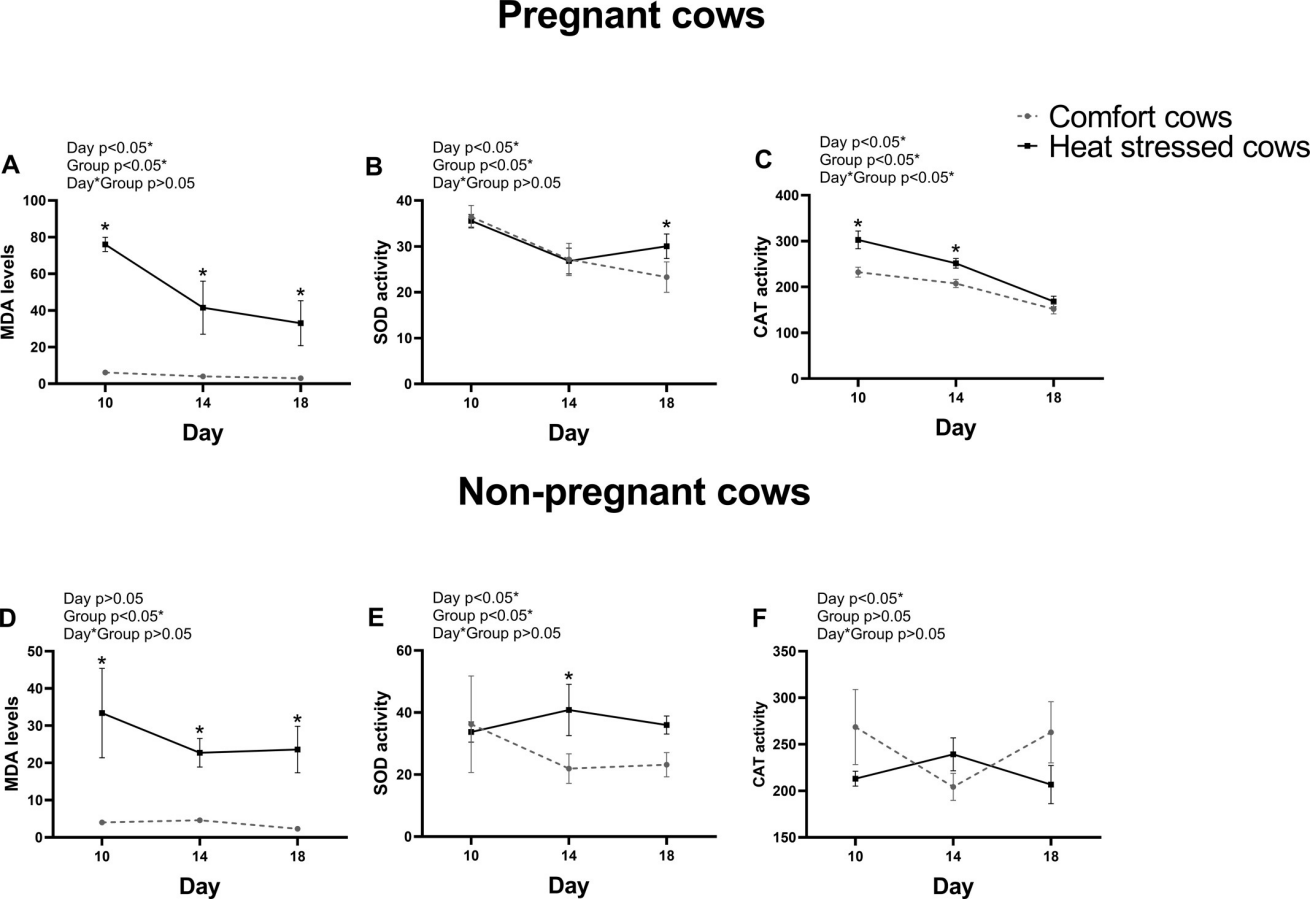
Oxidative stress-related parameters in blood of pregnant and non-pregnant cows in comfort or under heat stress conditions on days 10, 14 and 18 post-AI. A, B, and C represent pregnant cows; D, E, and F represent non-pregnant cows. A and D: MDA levels. B and E: SOD activity. C and F: CAT activity. Values are presented as mean ± S.E.M. The main effects of day, group and day by group interaction (day*group) are indicated. Asterisk represents difference at P<0.05 between comfort and heat stressed groups.

Oxidative Stress was evaluated using MDA concentration measurement in blood from cows under comfort or heat stress environment on Days 10, 14 and 18 post AI (Fig 2). In both pregnant and non-pregnant cows, MDA concentrations were greater (P<0.05) in heat stress environment on Days 10, 14 and 18. Pregnant heat stressed cows had Day 18 SOD activity and Day 10 and 14 CAT activity greater than comfort pregnant cows (P<0.05). Non-pregnant heat stressed cows had only Day 14 SOD activity greater than comfort non-pregnant cows (P<0.05). Greater MDA levels unbalanced with antioxidant enzymes in heat stressed cows indicate oxidative stress.

### ISGs expression in PMN from cows in comfort or under heat stress environment

**Fig 3:**
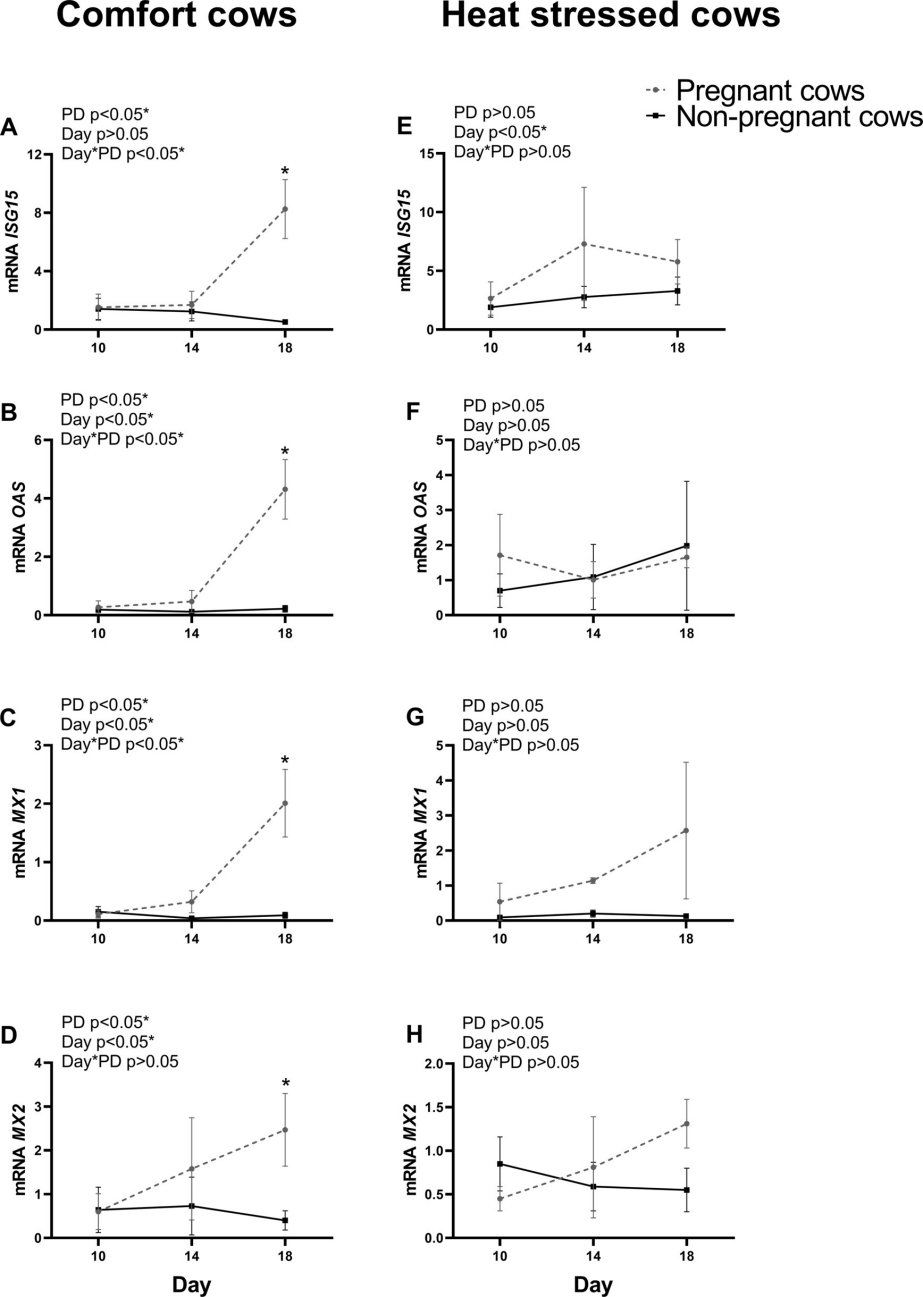
Interferon-stimulated genes expression in polymorphonuclear cells of pregnant and non-pregnant cows in comfort or under heat stress conditions on days 10, 14 and 18 post-AI. A-D represents ISGs of cows in comfort conditions; E-H represents ISGs of heat stressed cows. A and E: *ISG15;* B and F: *OAS;* C and G: *MX1;* D and H: *MX2*. Values are presented as mean ± S.E.M. The main effects of pregnancy diagnosis (PD), day and day by pregnancy diagnosis interaction (day*PD) are indicated. Asterisk represents difference at P<0.05 between pregnant and non-pregnant cows.

Relative mRNA expression of *ISG15*, *OAS*, *MX1* and *MX2* in PMN cells was evaluated in comfort or heat stressed cows on Days 10, 14 and 18 after AI (Fig 3). The expression of these genes was upregulated in Day 18 pregnant cows in comfort group when compared to non-pregnant (P<0.05; Fig 3A–3D). However, no difference between expression of all analyzed ISGs in non-pregnant and pregnant cows was observed when the cows were under heat stress (Fig 3E–3H).

### IFN pathway gene expression in PMN from cows in comfort or under heat stress environment

**Fig 4:**
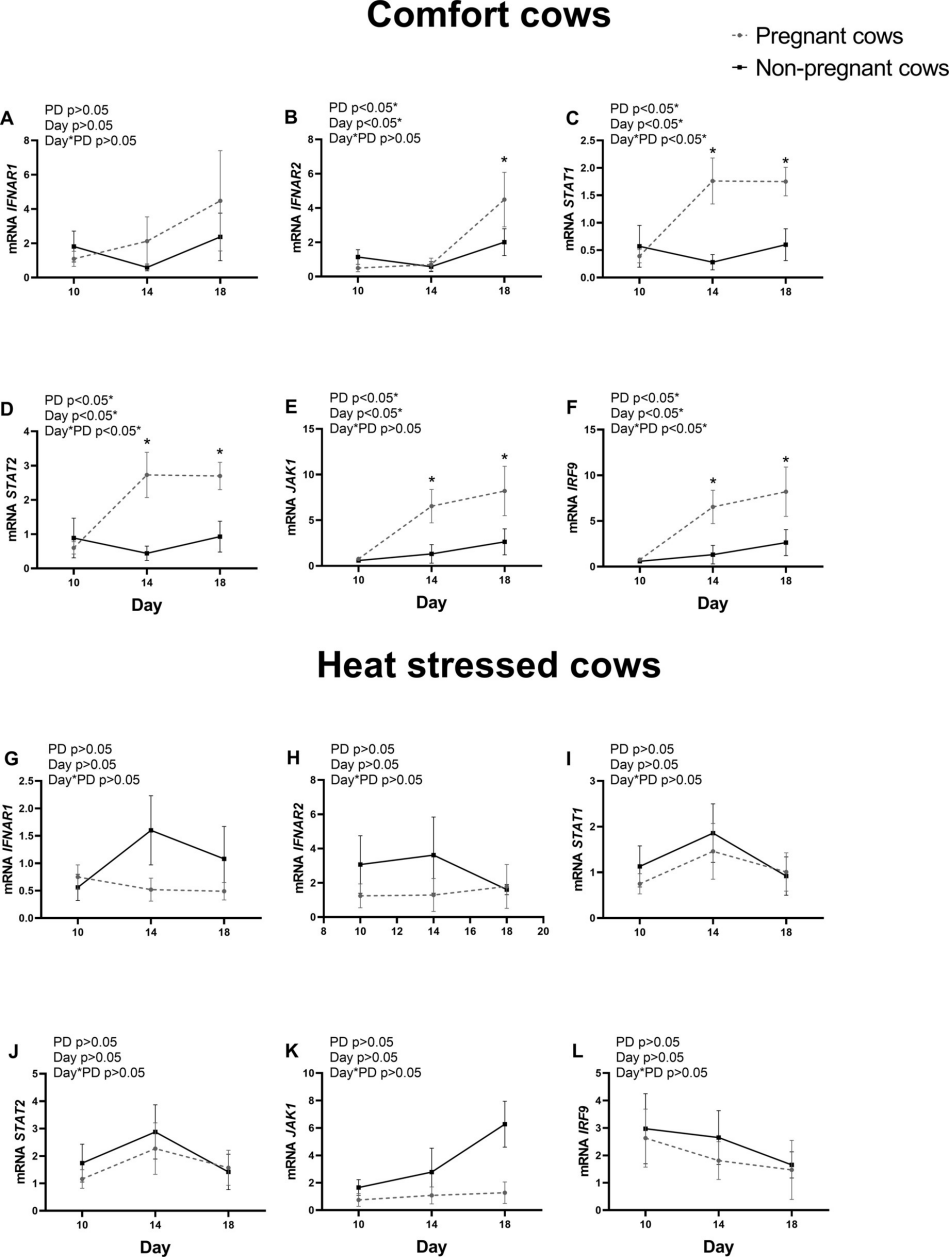
Interferon-pathway gene expression in polymorphonuclear cells of pregnant and non-pregnant cows on comfort or under heat stress conditions. A-F represents IFN-pathway components of cows in comfort conditions; G-L represents IFN-pathway components of heat stressed cows. A and G: *IFNAR1;* B and H: *IFNAR2;* C and I: *JAK1;* D and J: *STAT1;* E and K: *STAT2;* F and L: *IRF9*. Values are presented as mean ± S.E.M. The main effects of pregnancy diagnosis (PD), day and day by pregnancy diagnosis interaction (day*PD) are indicated. Asterisk represents difference at P<0.05 between pregnant and non-pregnant cows.

In order to identify IFN signaling, relative mRNA expression of *IFNAR1*, *IFNAR2*, *STAT1*, *STAT2*, *JAK1* and *IRF9* in PMN cells was evaluated in comfort or heat stressed cows on Days 10, 14 and 18 after AI (Fig 4). Besides *IFNAR1*, expression of IFN pathway genes in PMN was upregulated in pregnant but not in non-pregnant comfort group cows on Days 14 and 18 (P<0.05; Fig 4A–4F). However, expression of all evaluated IFN pathway genes was not different between pregnant and non-pregnant cows under heat stress (Fig 4G–4L).

### ISGs and IFN pathway expression in PMN of pregnant cows in comfort or under heat stress environment

**Fig 5:**
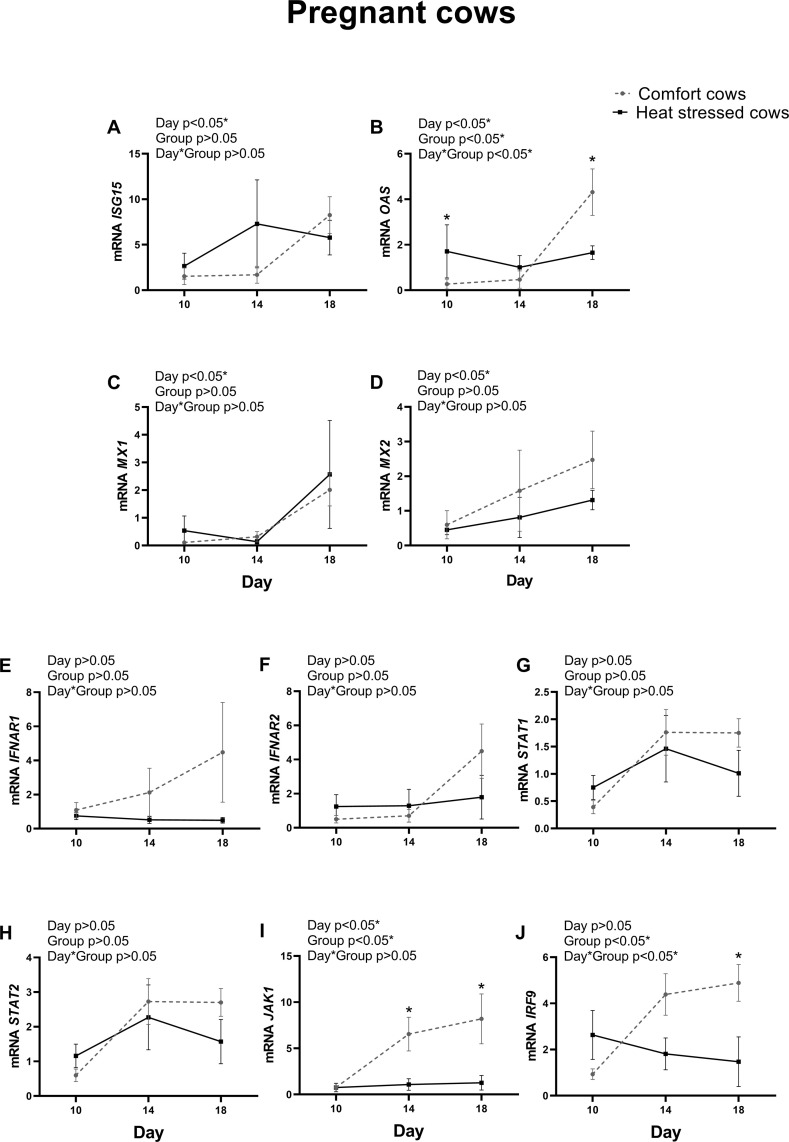
Interferon-stimulated genes and interferon-pathway gene expression in polymorphonuclear cells of pregnant cows in comfort or under heat stress conditions on days 10, 14 and 18 post-AI. A) *ISG15;* B) *OAS*; C) *MX1;* D) *MX2;* E) *IFNAR1;* F) *IFNAR2;* G) *STAT1;* H) *STAT2;* I) *JAK1;* J) *IRF9*. Values are presented as mean ± S.E.M. The main effects of day, group and day by group interaction (day*group) are indicated. Asterisk represents difference at P<0.05 between comfort and heat stressed groups.

For the purpose of identifying differences in pregnant cows, relative mRNA expression of ISGs and IFN pathway genes in PMN cells was compared only in pregnant cows in comfort or heat stressed environment on Days 10, 14 and 18 after AI (Fig 5). Among the ISGs, only *OAS* (Fig 5B) was greater (P<0.05) on Day 18 in PMN of comfort cows when compared to heat-stressed pregnant cows. When IFN pathway was analyzed, only *JAK1* (Fig 5I) was greater on Days 14 and 18 and *IRF9* (Fig 5J) (P<0.05) on Day 18 was greater in comfort group pregnant animals vs heat-stressed pregnant animals. All other genes were not different between pregnant cows in comfort or heat stressed environment. The analysis performed in non-pregnant cows is shown in S3 Fig.

## Discussion

In order to study the influence of heat stress on early pregnancy in cows, we designed and validated experimental model, allowing to evaluate effect of cold and warm season of the year on estrous occurrence and pregnancy rate, THI, RT, HR, RR, and daily milk yield. The experimental design allowed us to discover the following relevant findings: 1) CL diameter did not differ between comfort and heat stressed cows; however, the progesterone production was lower in pregnant heat stressed cows; 2) MDA levels were greater in both non-pregnant and pregnant cows under heat stress, while activity of the anti-oxidant enzymes SOD and CAT did not have the proportional increase, indicating oxidative stress in cows of both groups; 3) expression of ISGs and type I IFN pathway genes in neutrophils of comfort pregnant cows was increasing over time and reached a peak on Day 18, while non-pregnant cows maintained lower expression; 4) the expression pattern of ISGs and type I IFN pathway in neutrophils from heat stressed cows did not differ between non-pregnant and pregnant cows on all days.

Pregnant cows in comfort ambient environment display increased gene expression of genes of the type I IFN signaling pathway along with the expression of ISGs in time dependent manner, indicating upregulation of the pathway, while activation of the type I IFN pathway was not detected in pregnant heat stressed cows. Neutrophils are known to be the first response in the inflammatory process; however, it has been proposed that they could respond modulating local innate and adaptative immune system [42]. IFNT regulates expression of genes of the innate immune system in the uterus and also in peripheral immune cells and other tissues throughout the body [43]. The results presented here indicate that the embryo via secretion of INT activates the neutrophils responses only in comfort pregnant cows.

The environmental conditions seem to affect the maternal recognition of pregnancy signaling. Previous in vitro study from our group, demonstrated the influence of heat stress on oxidative stress and IFNT production [44]. Heat stressed pregnant cows did not have the same increased expression of interferon stimulated and IFN I pathway genes on Day 18 as it was found in cows in comfort temperature. Also, our data revealed that oxidative stress may be involved in progesterone production and expression of ISGs and IFN pathway, whereas MDA concentration was increased only in cows of heat stressed group on all experimental days. Notably, upregulation of genes directly related to maternal recognition of pregnancy was detected in PMN in dairy cows, which can provide insight into development of a new method to diagnose pregnancy.

Progesterone is the key hormone controlling early pregnancy [45] and its low concentration in early pregnancy period has been correlated to negative effects on embryo development and elongation [46]. Heat stress or increased metabolic rate reduce progesterone level in high daily milk yield cows [47–49]. Furthermore, the decrease of concentration of progesterone also can be associated with oxidative stress. It has been shown that long-term moderate oxidative stress reduces the potential for fertility. This effect may be due to poor follicular quality and consequently decreased progesterone [50]. It is reasonable to suggest that the reduction of concentration of progesterone without the decrease of CL diameter in pregnant cows under heat stress are due to oxidative stress present in these cows.

The increased SOD and CAT activities maintain low levels of MDA in pregnant cows in the comfort group; while in the heat stressed pregnant cows increased SOD and CAT activity is not able to prevent the increase of MDA level, indicating oxidative stress. It is known that exposure to heat stress results in higher mitochondrial and plasma levels of MDA, the major product of lipid peroxidation [10] and oxidative cellular stress [51, 52]. It has even been shown that MDA can be used as a blood marker for oxidative status of dairy cows during warm season [52]. Although studies show increase of antioxidant enzymes in hyperthermia situations [10], there is a study showing the decrease in SOD and CAT enzymes, which resulted in a significant reduction in thermal resistance [53]. The antioxidant enzymatic process was apparently not effective in cows under heat stress in our study. This condition seems to characterize a deficient antioxidant enzymatic system in heat stressed cows.

There are many genes upregulated by IFNT in early pregnancy and among all ISGs, we can highlight *ISG15*, *MX1*, *MX2* and *OAS* [18, 22] because they have greater expression in neutrophils, compared to other fractions of peripheral blood leucocytes [23]. In general, the amount of IFNT in the bloodstream is low and thus is difficult to detect, but IFNT activity can be detected in the bloodstream using radio immune assay [54] and antiviral assay [19, 21]. Another method to detect IFNT-response in the bloodstream is to identify ISGs gene expression, demonstrating the expressions of ISGs as IFNT endpoint activity. There are several studies that showed correlation between ISGs expression in peripheral blood leukocytes (PBL) during early pregnancy [22–24, 26].

Interestingly, we observed that *ISG15*, *OAS*, *MX1* and *MX2* genes were upregulated in PMN from pregnant cows in comfort group on Day 18 following AI, but not in heat stressed pregnant cows. One study demonstrated that heat stressed pregnant cows have greater ISGs expression [55], however, the THI in stressed cows in the study were lower than in cows in our study. The occurrence of heat stress with higher humidity, as in our study, lead to THI above 80, promoting a subtle increase in the expression of ISGs in stressed cows. The possible explanation for this observation could be that the embryonic cells that are responsible for production and secretion of IFNT at the beginning of the embryonic development [56, 57] were in oxidative stress. This is important because IFNT begins to be significantly expressed on Day 7 of development [58] and its peak production occurs between days 18 and 20 following conception [59] for the maternal recognition of pregnancy.

Based on the upregulation of ISGs by IFNT in PMN leukocytes, we investigated the type I IFN signaling pathway in PMN cells of non-pregnant and pregnant cows, in comfort or under heat stress. As expected, the *IFNAR2* receptor, *JAK1*, *STAT1* and *STAT2* cascade and *IRF9* regulatory factor were upregulated on Days 14 and 18 following AI in pregnant cows in comfort; however, no difference was observed in all IFN pathway genes of pregnant cows under heat stress. The increase of ISGs in PMN from pregnant cows only on Days 14 and 18 may be explained by the fact that the embryo did not start to elongate before Day 10, and, consequently, there is not enough amount of IFNT leaving the uterus at this time [60]. IFNT was found to modulate IFNAR2 subunit [23], and our in vivo data demonstrate upregulated IFNAR2 but not IFNAR1 in PMN from cows in comfort. This suggests the receptor subunit controlled by IFNT is IFNAR2. Pregnant cows under heat stress conditions did not show the same pattern of ISGs and IFN pathway gene expression when compared to pregnant comfort cows. Although, when we compared pregnant cows in comfort to heat stressed cows, there were no differences in ISGs and IFN pathway gene expression. We believe that oxidative stress not only decreases concentration of progesterone, but also impairs IFN gene pathway and ISGs expression, as well as activation of interferon-primed neutrophils. One study characterized genes and pathways that respond to heat stress in Holstein calves, where the transcriptome analysis showed that expression of genes such as IFNAR2 and STATs is increased in response to heat stress [61]. Another study reported that JAKs are redox-sensitive enzymes [62]. These findings support our hypothesis that cows under influence of heat and oxidative stress, even if they are pregnant, have a distinct response regarding to IFNT endocrine signaling in PMNs. This response makes it difficult to accurately use expression of these ISGs to identify early pregnancy. In addition to the effects of HS on IFNT signaling [44], IFNT has been shown to modulate local and systemic innate immune response, carried out mainly by neutrophils [43, 63]. Neutrophils are essential for innate immunity and resistance to pathogens, not only acting as a final effector of an acute inflammatory response, but also secreting factors such as cytokines to activate cells of innate and adaptative immune response. They respond to tissue- and cell-derived signal undergoing polarization [42]. This led us to consider that IFNT and other embryokines may activate circulating neutrophils, required in early pregnancy to protect the mother as a first defense barrier against a viral infection, as shown by Shoemaker, Smirnova [64]; And this may be impaired in stressed (heat and/or oxidative) environments. Exactly how HS and IFNT modulate local and systemic immune response through early pregnancy remains unclear.

**Fig 6:**
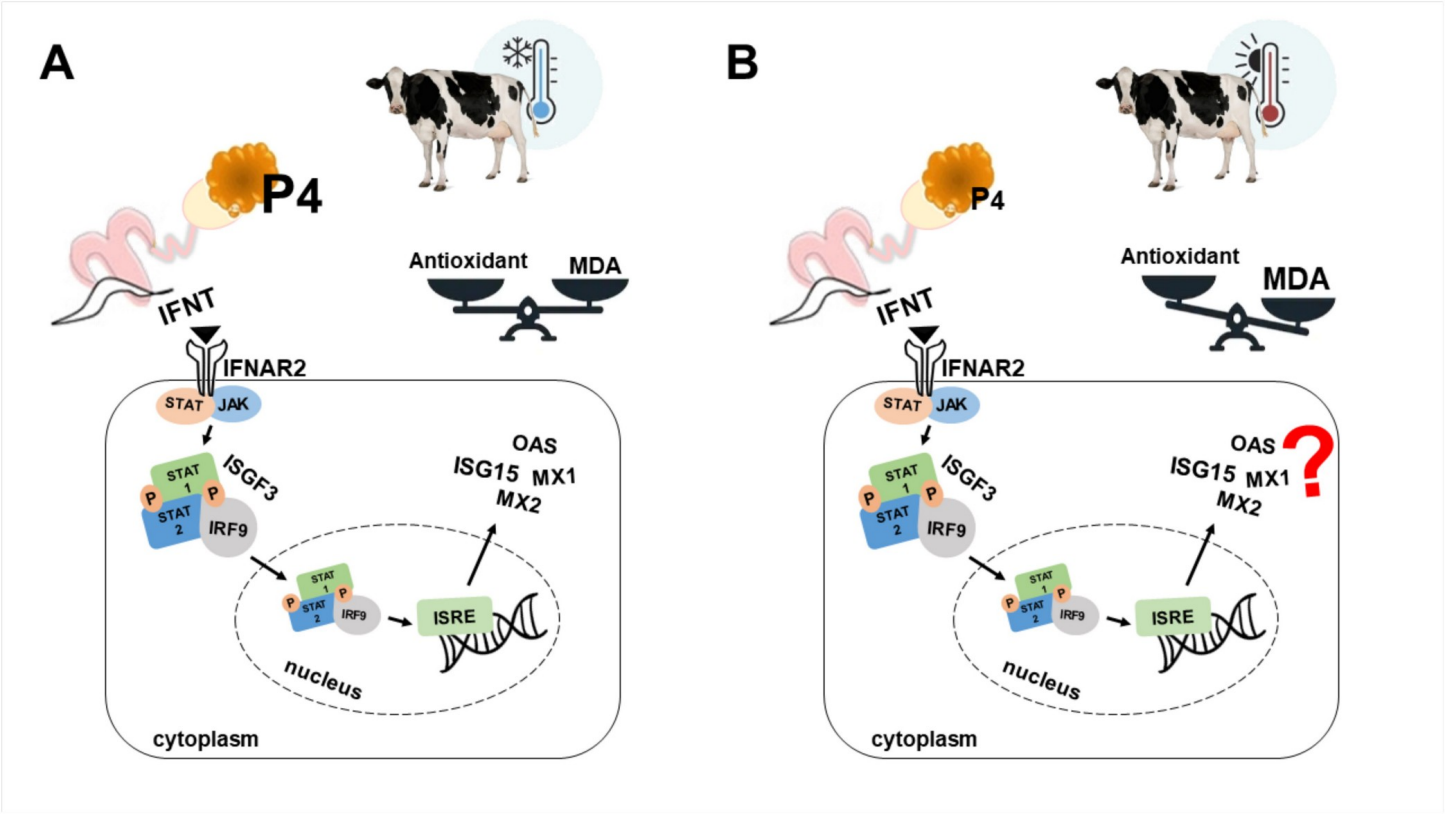
Schematic model illustrating the main conclusions. A) Cows in comfort environment maintains oxidative balance regardless of pregnancy status. Type I IFN pathway and ISGs genes were upregulated on Day 18 in PMN cells of pregnant cows. However, B) Heat stress conditions induce oxidative stress in pregnant and non-pregnant cows. In pregnant cows, HS decreases progesterone concentration and impairs ISGs and Type I IFN pathway gene expression in PMN cells.

In conclusion, the present study observed that oxidative stress caused by heat stress decreases progesterone production and alters ISGs and IFN pathway gene expression in PMN cells of dairy cows in early pregnancy (Fig 6). The heat stress in pregnant cows not only impairs the ISGs gene expression but also interferes with IFN pathway activation, possibly modifying systemic innate immune response. Lower conception rates in cows under heat stress are influenced by several factors, and the unbalanced oxidative stress associated with impaired IFN pathway activation influencing innate immunity response could be one of the main contributing factors.

## Supporting information

S1 Table Temperature-humidity index (THI) calculated on experimental days on two different seasons. The samples from cows of the comfort group were collected on late winter/early spring and the samples from the cows of heat stressed group were collected on summer.
(TIF)

S1 Fig Effect of season on rectal temperature (RT), heart rate (HR) and respiratory rate (RR) in cows on comfort or under heat stress conditions. RT, HR and RR were measured on Days 10, 14 and 18 after AI. A) RT (°C) was measured with a large animal clinical thermometer inserted to depth of 3 cm into the animal rectum and held to maintain contact with the mucosa for one minute. B) HR was expressed in beats per minute (bpm) and was obtained using a flexible stethoscope placed directly into the left thoracic region under one of the auscultation foci for 30 seconds, multiplied by 2 to obtain the number of heart beats per minute. C) RR was expressed in breaths per minute (bpm) and was obtained using a timer to count the flank movements of the animal for 30 seconds, multiplied by 2 to obtain the number of breaths per minute. Values are presented as mean ± S.E.M. Asterisk represent significance at p<0.05 between comfort and heat stressed cows.
(TIF)

S2 Fig Effect of season on productive performance of dairy cows on comfort or under heat stress conditions. A) Daily milk yield (kg) was measured during the two milking on AI day of each animal. B) Days in milk started on the last calving until AI day. Values are presented as mean ± S.E.M. Asterisk represent significance at p<0.05 between comfort and heat stressed cows groups.
(TIF)

S3 Fig Interferon-stimulated genes and interferon-pathway gene expression in polymorphonuclear cells on days 10, 14 and 18 post-AI of non-pregnant cows compared on comfort or under heat stress conditions. A) *ISG15*. B) *OAS*. C) *MX1*. D) *MX2*. E) *IFNAR1*. F) *IFNAR2*. G) *STAT1*. H) *STAT2*. I) *JAK1*. J) *IRF9*. Values are presented as mean ± S.E.M. The main effects of day, group and day by group interaction (day*group) are indicated. Asterisk represent difference at p<0.05 between comfort and heat stressed groups.
(TIF)
