# Supplementary figures and images for: Heat stress modulates polymorphonuclear cell response in early pregnancy cows: I. interferon pathway and oxidative stress
Source: PLoS One. 2021 Sep 20;16(9):e0257418. doi: 10.1371/journal.pone.0257418 (PMC8452032; doi:10.1371/journal.pone.0257418)

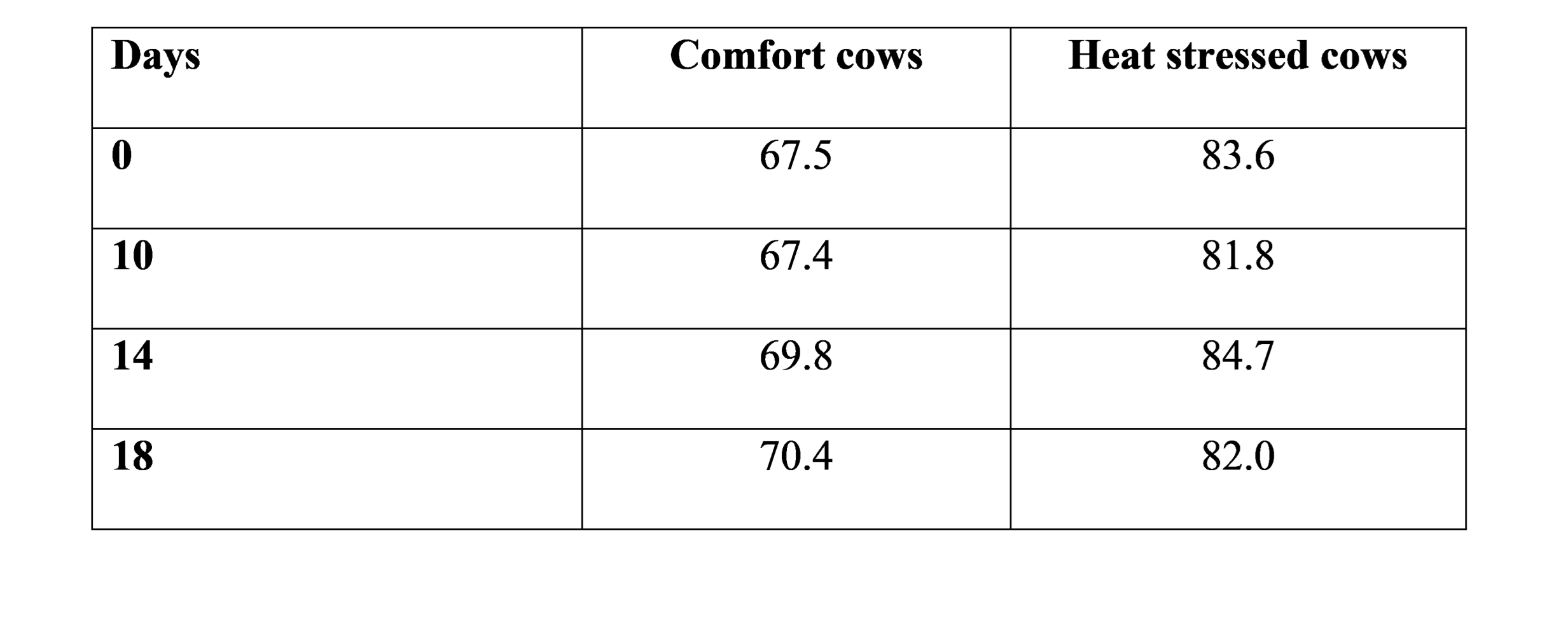

Supplement: S1 Table — The samples from cows of the comfort group were collected on late winter/early spring and the samples from the cows of heat stressed group were collected on summer. (TIF) [file pone.0257418.s001.tif]

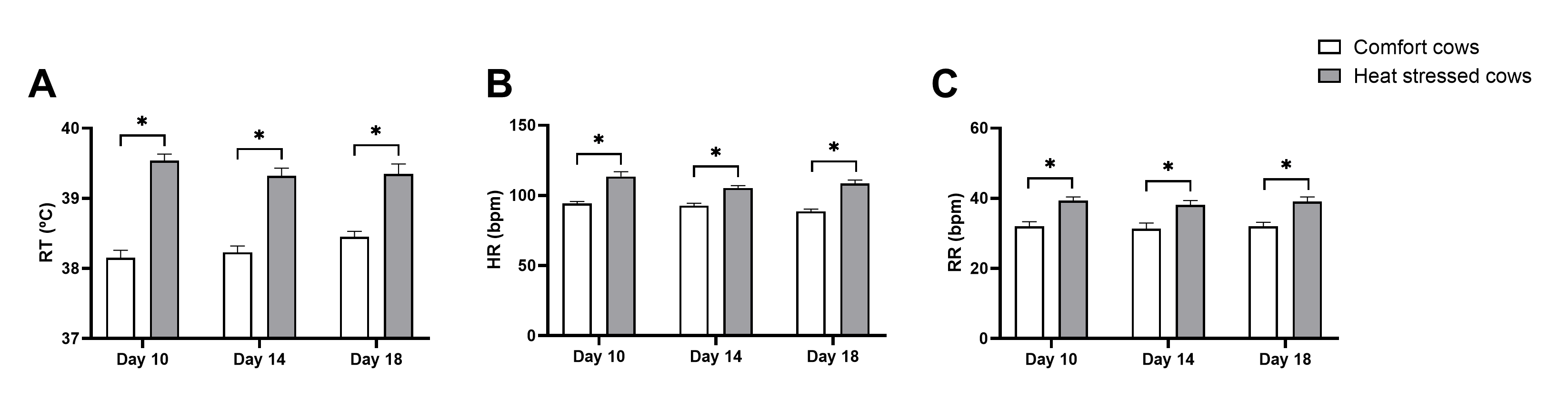

Supplement: S1 Fig — RT, HR and RR were measured on Days 10, 14 and 18 after AI. A) RT (°C) was measured with a large animal clinical thermometer inserted to depth of 3 cm into the animal rectum and held to maintain contact with the mucosa for one minute. B) HR was expressed in beats per minute (bpm) and was obtained using a flexible stethoscope placed directly into the left thoracic region under one of the auscultation foci for 30 seconds, multiplied by 2 to obtain the number of heart beats per minute. C) RR was expressed in breaths per minute (bpm) and was obtained using a timer to count the flank movements of the animal for 30 seconds, multiplied by 2 to obtain the number of breaths per minute. Values are presented as mean ± S.E.M. Asterisk represent significance at p<0.05 between comfort and heat stressed cows. (TIF) [file pone.0257418.s002.tif]

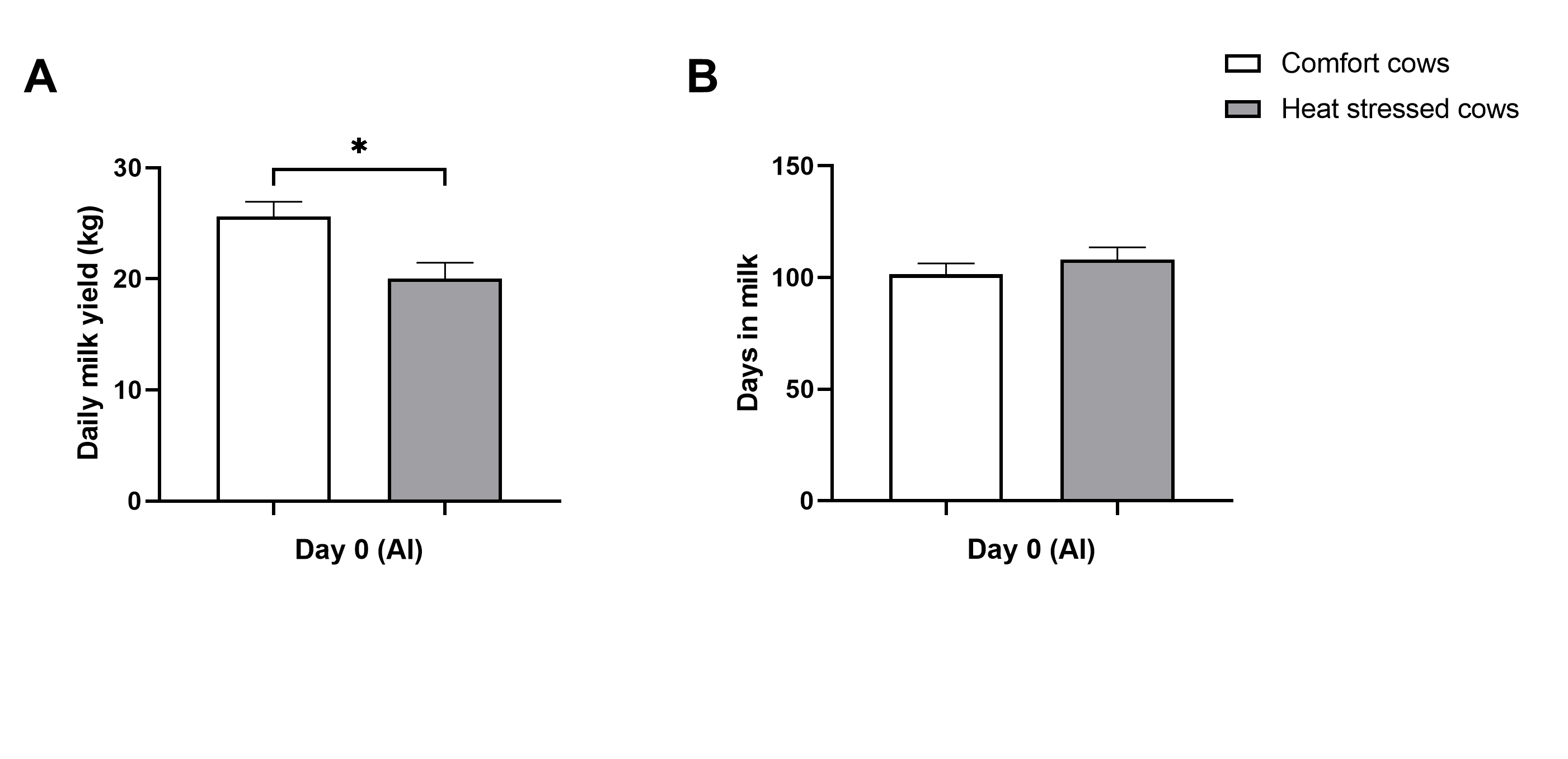

Supplement: S2 Fig — A) Daily milk yield (kg) was measured during the two milking on AI day of each animal. B) Days in milk started on the last calving until AI day. Values are presented as mean ± S.E.M. Asterisk represent significance at p<0.05 between comfort and heat stressed cows groups. (TIF) [file pone.0257418.s003.tif]

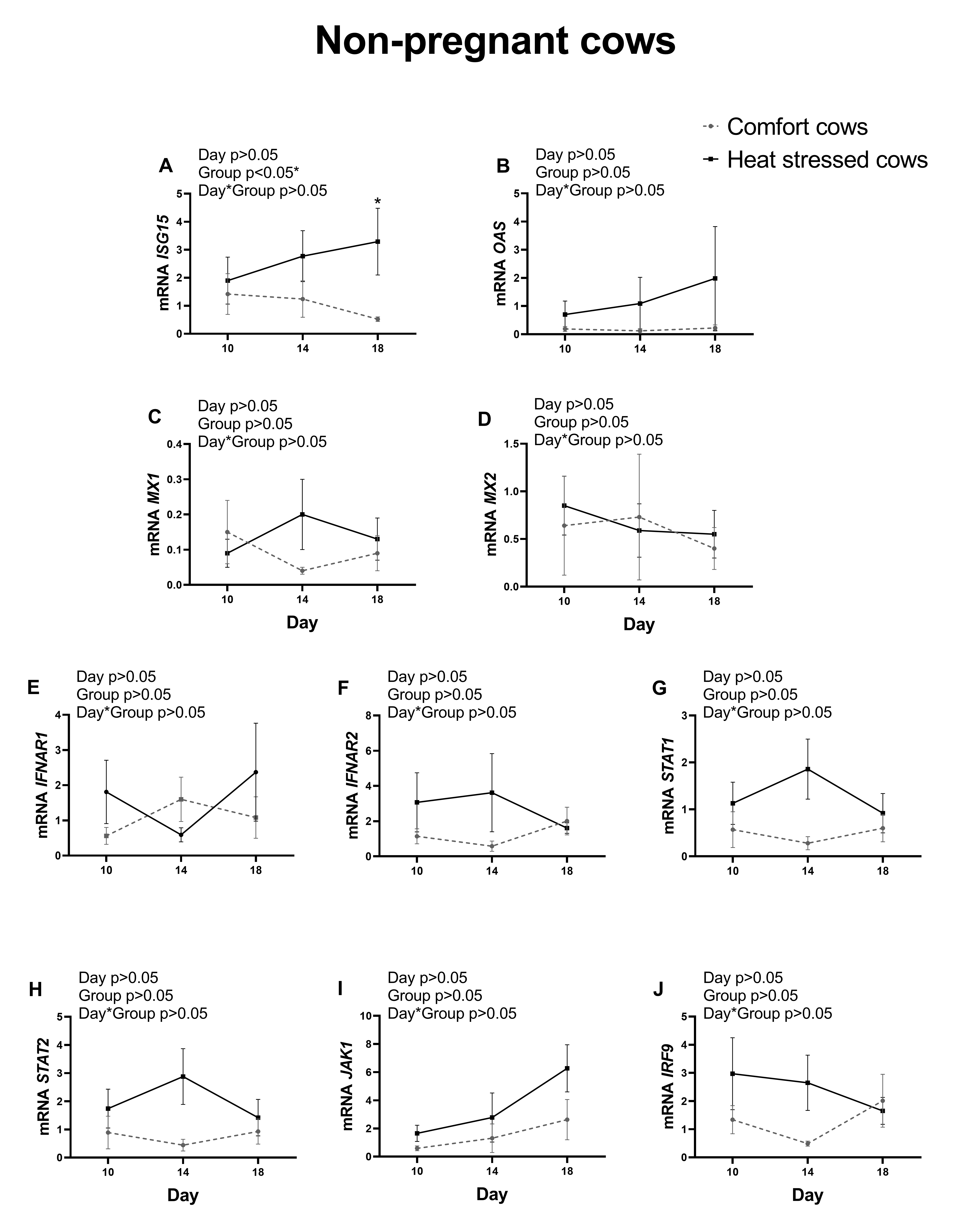

Supplement: S3 Fig — A) ISG15. B) OAS. C) MX1. D) MX2. E) IFNAR1. F) IFNAR2. G) STAT1. H) STAT2. I) JAK1. J) IRF9. Values are presented as mean ± S.E.M. The main effects of day, group and day by group interaction (day*group) are indicated. Asterisk represent difference at p<0.05 between comfort and heat stressed groups. (TIF) [file pone.0257418.s004.tif]
